# Supplementary material for: Fixation patterns in pairs of facial expressions—preferences of self-critical individuals
Source: PeerJ Comput Sci. 2024 Nov 1;10:e2413. doi: 10.7717/peerj-cs.2413 (PMC11623007; doi:10.7717/peerj-cs.2413)
Supplement: Supplemental Information 1 [file peerj-cs-10-2413-s001.docx]

**Appendix A**

Regression coefficients for predicting time to first fixation (TTFF_L and TTFF_R)

| Variable | B | 95% CI | β | T | P |
| --- | --- | --- | --- | --- | --- |
| *TTFF_L* | | | | | |
| IS | -.272 | [-.342, -.201] | -.085 | -7.561 | .000 |
| Emotion of the stimulus | .000 | [-.003, .003] | -.001 | -.089 | .929 |
| Age of the stimulus | -.005 | [-.048, .039] | -.002 | -.206 | .836 |
| Sex of the stimulus | .003 | [-.038, .103] | .010 | .906 | .365 |
| *TTFF_R* | | | | | |
| IS | -.271 | [-.342, -.200] | -.084 | -7.479 | .000 |
| Emotion of the stimulus | -.001 | [-.004, .002] | -.010 | -.856 | .392 |
| Age of the stimulus | .023 | [-.021, .066] | .012 | 1.034 | .301 |
| Sex of the stimulus | -.011 | [-.082, .060] | -.004 | -.314 | .753 |

Note TTFF_L R^2^ _adj_ = .007 (N = 7811, p = .000). TTFF_R R^2^ _adj_ = .007 (N = 7811, p = .000). CI = confidence interval for B.

**Appendix B**

Regression coefficients for predicting first fixation duration (FFD_L and FFD_R)

| Variable | B | 95% CI | β | T | p |
| --- | --- | --- | --- | --- | --- |
| *FFD_L* | | | | | |
| IS | .040 | [.238, .337] | .047 | 4.183 | .000 |
| Emotion of the stimulus | .000 | [-.001, .001] | -.005 | -.446 | .655 |
| Age of the stimulus | .005 | [-.006, .016] | .010 | .854 | .393 |
| Sex of the stimulus | .008 | [-.011, .026] | .009 | .795 | .427 |
| *FFD_R* | | | | | |
| IS | .036 | [-.016, .056] | .039 | 3.454 | .001 |
| Emotion of the stimulus | .000 | [-.001, .001] | -.003 | -.233 | .816 |
| Age of the stimulus | .005 | [-.007, .018] | .009 | .797 | .426 |
| Sex of the stimulus | .005 | [-.015, .026] | .006 | .501 | .616 |

Note FFD_L R^2^ _adj_ = .002 (N = 7811, p = .001). FFD_R R^2^ _adj_ = .001 (N = 7811, p = .012). CI = confidence interval for B.

**Appendix C**

Regression coefficients for predicting total fixation time (TFD_L and TFD_R)

| Variable | B | 95% CI | β | T | P |
| --- | --- | --- | --- | --- | --- |
| *TFD_L* | | | | | |
| IS | .112 | [.067, -.156] | .055 | 4.887 | .000 |
| Emotion of the stimulus | .000 | [-.002, .002] | .001 | .114 | .909 |
| Age of the stimulus | .002 | [-.025, .030] | .002 | .160 | .873 |
| Sex of the stimulus | -.005 | [-.050, .039] | -.003 | -.233 | .816 |
| *TFD_R* | | | | | |
| IS | .074 | [.028, .121] | .036 | 3.155 | .002 |
| Emotion of the stimulus | -.002 | [-.004, .000] | -.022 | -1.924 | .054 |
| Age of the stimulus | -.021 | [-.049, .008] | -.016 | -1.440 | .150 |
| Sex of the stimulus | .005 | [-.041, .051] | .002 | .208 | .836 |

Note TFD_L R^2^ _adj_ = .003 (N = 7811, p = .000). TFD_R R^2^ _adj_ = .002 (N = 7811, p = .003). CI = confidence interval for B.

**Appendix D**

Regression coefficients for predicting total fixation time in AOI as % of overall exposure time (TFD_ratio_L and TFD_ratio_R)

| Variable | B | 95% CI | β | T | p |
| --- | --- | --- | --- | --- | --- |
| *TFD_ratio_L* | | | | | |
| IS | 4.538 | [3.619, 5.458] | .109 | 9.676 | .000 |
| Emotion of the stimulus | .022 | [-.016, .060] | .013 | 1.152 | .249 |
| Age of the stimulus | .181 | [-.382, .744] | .007 | .630 | .529 |
| Sex of the stimulus | -.269 | [-1.118, .651] | -.006 | -.573 | .567 |
| *TFD_ratio_R* | | | | | |
| IS | 1.494 | [-.342, -.200] | .036 | .568 | 2.420 |
| Emotion of the stimulus | -.038 | [-.004, .002] | -.022 | -.076 | .001 |
| Age of the stimulus | -.410 | [-.977, .157] | -.016 | -1.419 | .156 |
| Sex of the stimulus | .103 | [-.083, 1.028] | .002 | .217 | .828 |

Note TFD_ratio_L R^2^ _adj_ = .012 (N = 7811, p = .000). TFD_ratio_R R^2^ _adj_ = .002 (N = 7811, p = .003). CI = confidence interval for B.

**Appendix E**

Regression coefficients for predicting average fixation duration (AFD_L and AFD_R)

| Variable | B | 95% CI | β | T | p |
| --- | --- | --- | --- | --- | --- |
| *AFD_L* | | | | | |
| IS | .049 | [.033, .065] | .069 | 6.084 | .000 |
| Emotion of the stimulus | .000 | [-.001, .001] | .001 | .102 | .919 |
| Age of the stimulus | .002 | [-.007, .012] | .005 | .458 | .647 |
| Sex of the stimulus | .015 | [-.001, .031] | .020 | 1.815 | .070 |
| *AFD_R* | | | | | |
| IS | .038 | [.021, .054] | .050 | 4.454 | .000 |
| Emotion of the stimulus | .000 | [-.001, .000] | -.011 | -.965 | .335 |
| Age of the stimulus | .005 | [-.005, .015] | .011 | .955 | .340 |
| Sex of the stimulus | .012 | [-.004, .029] | .016 | 1.445 | .148 |

Note AFD_L R^2^ _adj_ = .005 (N = 7811, p = .000). AFD_R R^2^ _adj_ = .003 (N = 7811, p = .000). CI = confidence interval for B.

**Appendix F**

Regression coefficients for predicting fixation count (FC_L and FC_R)

| Variable | B | 95% CI | β | T | p |
| --- | --- | --- | --- | --- | --- |
| *FC_L* | | | | | |
| IS | .627 | [.512, .742] | .120 | 10.693 | .000 |
| Emotion of the stimulus | .004 | [-.001, .008] | .016 | 1.445 | .148 |
| Age of the stimulus | -.008 | [-.078, .063] | -.002 | -.211 | .833 |
| Sex of the stimulus | -.142 | [-.257, -.027] | -.027 | -2.428 | .015 |
| *FC_R* | | | | | |
| IS | .294 | [-.185, -.403] | .060 | 5.278 | .000 |
| Emotion of the stimulus | .000 | [-.005, .004] | .000 | -.030 | .976 |
| Age of the stimulus | -.064 | [-.130, .003] | -.021 | -1.866 | .062 |
| Sex of the stimulus | -.091 | [-.200, .018] | -.018 | -1.634 | .102 |

Note FC_L R^2^ _adj_ = .015 (N = 7811, p = .000). FC_R R^2^ _adj_ = .004 (N = 7811, p = .000). CI = confidence interval for B.

**Appendix G**

Regression coefficients for predicting time to first fixation (TTFF_L and TTFF_R)

| Variable | B | 95% CI | β | T | P |
| --- | --- | --- | --- | --- | --- |
| *TTFF_L* | | | | | |
| HS | .002 | [-.058, .062] | .001 | .070 | .945 |
| Emotion of the stimulus | .000 | [-.003, .002] | -.001 | -.115 | .908 |
| Age of the stimulus | -.022 | [-.058, .015] | -.012 | -1.166 | .244 |
| Sex of the stimulus | .030 | [-.029, .089] | .010 | .993 | .321 |
| *TTFF_R* | | | | | |
| HS | .001 | [-.067, .069] | .000 | .026 | .979 |
| Emotion of the stimulus | -.001 | [-.003, .002] | -.005 | -.482 | .630 |
| Age of the stimulus | -.002 | [-.043, .040] | -.001 | -.084 | .933 |
| Sex of the stimulus | .015 | [-.053, .082] | .004 | .427 | .669 |

Note TTFF_L R^2^ _adj_ = .000 (N = 9317, p = .670). TTFF_R R^2^ _adj_ = .000 (N = 9317, p = .981). CI = confidence interval for B.

**Appendix H**

Regression coefficients for predicting first fixation duration (FFD_L and FFD_R)

| Variable | B | 95% CI | β | T | p |
| --- | --- | --- | --- | --- | --- |
| *FFD_L* | | | | | |
| HS | .007 | [-.011, .026] | .008 | 0.779 | .436 |
| Emotion of the stimulus | .000 | [-.001, .001] | .000 | -.027 | .979 |
| Age of the stimulus | .000 | [-.011, .012] | .001 | .068 | .946 |
| Sex of the stimulus | .007 | [-.011, .025] | .008 | .785 | .433 |
| *FFD_R* | | | | | |
| HS | .009 | [-.010, .028] | .010 | .927 | .354 |
| Emotion of the stimulus | .000 | [-.001, .001] | -.006 | -.552 | .581 |
| Age of the stimulus | .006 | [-.005, .018] | .009 | .797 | .426 |
| Sex of the stimulus | .005 | [-.015, .026] | .011 | 1.045 | .296 |

Note FFD_L R^2^ _adj_ = .000 (N = 9317, p = .874). FFD_R R^2^ _adj_ = .000 (N = 9317, p = .546). CI = confidence interval for B.

**Appendix I**

Regression coefficients for predicting total fixation time (TFD_L and TFD_R)

| Variable | B | 95% CI | β | T | p |
| --- | --- | --- | --- | --- | --- |
| *TFD_L* | | | | | |
| HS | -.017 | [-.059, .025] | -.008 | -.776 | .438 |
| Emotion of the stimulus | .001 | [-.001, .003] | .010 | .997 | .319 |
| Age of the stimulus | -.015 | [-.041, .010] | -.012 | -1.186 | .236 |
| Sex of the stimulus | -.013 | [-.055, .028] | -.007 | -.628 | .530 |
| *TFD_R* | | | | | |
| HS | -.088 | [-.131, -.045] | -.042 | -4.028 | .000 |
| Emotion of the stimulus | -.001 | [-.003, .000] | -.015 | -1.442 | .149 |
| Age of the stimulus | -.019 | [-.045, .007] | -.015 | -1.461 | .144 |
| Sex of the stimulus | -.007 | [-.049, .035] | -.003 | -.325 | .745 |

Note TFD_L R^2^ _adj_ = .000 (N = 9317, p = .493). TFD_R R^2^ _adj_ = .002 (N = 9317, p = .000). CI = confidence interval for B.

**Appendix J**

Regression coefficients for predicting total fixation time in AOI as % of overall exposure time (TFD_ratio_L and TFD_ratio_R)

| Variable | B | 95% CI | β | T | p |
| --- | --- | --- | --- | --- | --- |
| *TFD_ratio_L* | | | | | |
| HS | .574 | [-.285, 1.433] | .014 | 1.310 | .190 |
| Emotion of the stimulus | .034 | [-.001 .069] | .020 | 1.912 | .056 |
| Age of the stimulus | -.091 | [-.612, .430] | -.004 | -.341 | .733 |
| Sex of the stimulus | -.645 | [-1.496, .206] | -.015 | -1.485 | .138 |
| *TFD_ratio_R* | | | | | |
| HS | -1.749 | [-2.603, -.894] | -.042 | -4.012 | .000 |
| Emotion of the stimulus | -.026 | [-.061, .009] | -.015 | -1.455 | .146 |
| Age of the stimulus | -.384 | [-.903, .134] | -.015 | -1.453 | .146 |
| Sex of the stimulus | -.141 | [-.988, .705] | -.003 | -.327 | .743 |

Note TFD_ratio_L R^2^ _adj_ = .000 (N = 9317, p = .104). TFD_ratio_R R^2^ _adj_ = .002 (N = 9317, p = .000). CI = confidence interval for B.

**Appendix K**

Regression coefficients for predicting average fixation duration (AFD_L and AFD_R)

| Variable | B | 95% CI | β | T | p |
| --- | --- | --- | --- | --- | --- |
| *AFD_L* | | | | | |
| HS | .015 | [.000, .031] | .020 | 1.908 | .056 |
| Emotion of the stimulus | .000 | [-.001, .001] | .003 | .333 | .739 |
| Age of the stimulus | .002 | [-.008, .012] | .004 | .397 | .691 |
| Sex of the stimulus | .012 | [-.004, .028] | .015 | 1.492 | .136 |
| *AFD_R* | | | | | |
| HS | .001 | [-.015, .017] | .001 | .098 | .922 |
| Emotion of the stimulus | .000 | [-.001, .000] | -.011 | -1.102 | .271 |
| Age of the stimulus | .007 | [-.003, .016] | .014 | 1.342 | .180 |
| Sex of the stimulus | .008 | [-.008, .024] | .011 | 1.014 | .311 |

Note AFD_L R^2^ _adj_ = .000 (N = 93171, p = .189). AFD_R R^2^ _adj_ = .000 (N = 9317, p = .398). CI = confidence interval for B.

**Appendix L**

Regression coefficients for predicting fixation count (FC_L and FC_R)

| Variable | B | 95% CI | β | T | p |
| --- | --- | --- | --- | --- | --- |
| *FC_L* | | | | | |
| HS | .090 | [-.017, .198] | .017 | 1.647 | .099 |
| Emotion of the stimulus | .005 | [.000, .009] | .022 | 2.138 | .033 |
| Age of the stimulus | -.012 | [-.078, .053] | -.004 | -.376 | .707 |
| Sex of the stimulus | -.158 | [-.264, -.051] | -.030 | -2.909 | .033 |
| *FC_R* | | | | | |
| HS | -.185 | [-.289, -.082] | -.036 | -3.508 | .000 |
| Emotion of the stimulus | .000 | [-.004, .005] | .002 | .188 | .851 |
| Age of the stimulus | -.069 | [-.132, .007] | -.022 | -2.166 | .030 |
| Sex of the stimulus | -.055 | [-.158, .048] | -.011 | -1.052 | .293 |

Note FC_L R^2^ _adj_ = .001 (N = 9317, p = .003). FC_R R^2^ _adj_ = .002 (N = 9317, p = .001). CI = confidence interval for B.
